# A Case of Diversion Colitis Successfully Treated by Synbiotic Stimulation via the Efferent Limb

**DOI:** 10.1155/cris/4985474

**Published:** 2026-10-21

**Authors:** Yasuhito Tsubokawa, Kazuhiro Nishida, Takaharu Kato

**Affiliations:** ^1^ Department of Gastrointestinal Surgery Higashi Saitama General Hospital Satte Saitama Japan

**Keywords:** diversion colitis, efferent limb, ileostomy closure, postoperative ileus, probiotics, protective ileostomy, stimulation

## Abstract

Diversion colitis (DC) is an inflammatory condition that develops in a bowel segment excluded from the fecal stream after colostomy or ileostomy. We report a case of symptomatic DC successfully treated with synbiotic stimulation via the efferent limb of an ileostomy. A 75‐year‐old man underwent endoscopic mucosal resection (EMR) for a transverse colon polyp. During the procedure, thermal injury caused a perforation at the splenic flexure, necessitating emergency partial resection of the transverse colon, including the perforated site, and creation of an ileostomy. Two weeks after surgery, the patient developed recurrent hematochezia. Colonoscopy revealed severe mucosal atrophy and friability predominantly in the excluded right colon, leading to a diagnosis of DC. Because the inflammatory changes were more prominent in the right colon, synbiotic stimulation was administered anterogradely via the efferent limb of the stoma. Follow‐up colonoscopy performed 1 month later demonstrated marked improvement in the mucosal findings. The patient subsequently underwent stoma closure, and the postoperative course was uneventful. This case suggests that synbiotic stimulation via the efferent limb may be an effective treatment option for symptomatic DC and may improve the condition of the excluded bowel before stoma closure.

## 1. Introduction

Diversion colitis (DC) was first described by Glotzer et al. [1] as an inflammatory condition that develops in a bowel segment excluded from the fecal stream following colostomy or ileostomy. The pathogenesis of DC is thought to involve a deficiency of intraluminal nutritional factors, particularly short‐chain fatty acids (SCFAs), as well as alterations in the intestinal microbiota [2]. Although most patients remain asymptomatic, some develop symptoms such as hematochezia and abdominal pain [3]. Current treatment options include SCFA enemas, steroid enemas, fecal microbiota transplantation, and restoration of intestinal continuity by stoma closure [2, 4, 5].

Recent studies have suggested that preoperative physiological stimulation, including synbiotic therapy, may reduce the incidence of postoperative ileus (POI), the most common complication following stoma closure [6]. However, few studies have evaluated its effect on more serious postoperative complications, such as anastomotic leakage.

To our knowledge, only a limited number of cases have described synbiotic stimulation administered through the distal limb of a stoma. Herein, we report a case of DC diagnosed after the onset of hematochezia that was successfully treated with synbiotic stimulation via the distal limb of the stoma, suggesting this approach may represent a useful therapeutic option for symptomatic DC.

## 2. Case Report

A 75‐year‐old man receiving edoxaban tosilate hydrate for atheroembolic cerebral infarction presented with a 4‐month history of hematochezia.

Colonoscopy revealed a 10‐mm type Is (sessile) polyp in the transverse colon, and endoscopic mucosal resection (EMR) was performed. Persistent bleeding from the resection site required repeated electrocautery for hemostasis.

Two days after EMR, the patient developed abdominal pain accompanied by a decreased body temperature of 35.6°C. Laboratory findings showed an elevated white blood cell (WBC) count of 15,000/μL and a C‐reactive protein (CRP) level of 14 mg/dL, indicating significant inflammation. Contrast‐enhanced CT demonstrated increased pericolic fat density and extraluminal free air around the EMR site, suggesting colonic perforation caused by thermal injury (Figure 1). Emergency partial resection of the transverse colon, including the perforated area, and creation of a double‐barrel ileostomy were performed on the same day.

**Figure 1:**
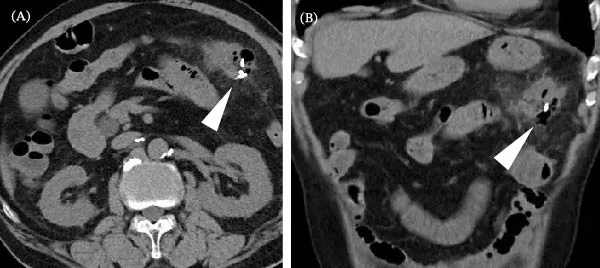
CT findings after EMR in the transverse colon. (A) Axial CT image showing increased fat stranding and extraluminal free air around the EMR site. (B) Coronal CT image demonstrating the same findings.

Histopathological examination revealed a 30 mm × 38 mm full‐thickness defect with subserosal abscess formation (Figure 2). The resected polyp was an adenoma containing intramucosal carcinoma, and the resection margins were negative.

**Figure 2:**
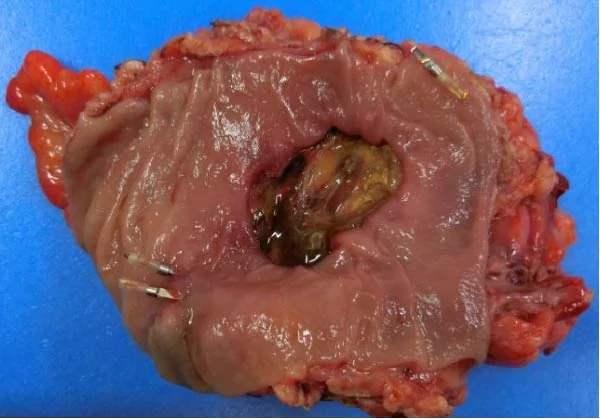
Histopathological findings after surgery. The perforated area measured 30 mm × 38 mm, with complete loss of the mucosa and muscularis propria. A subserosal abscess was also observed.

Because postoperative hematochezia persisted, colonoscopy was performed on postoperative day (POD) 13 after ileostomy creation to identify the source of bleeding and evaluate the anastomosis. Although no evidence of anastomotic leakage or bleeding was found, a large amount of blood clot was detected in the proximal bowel to the anastomosis.

Repeat colonoscopy performed on POD41 after ileostomy creation revealed marked mucosal atrophy and friability predominantly in the right‐sided excluded colon (Figure 3A). These inflammatory changes were confined to the diverted bowel segment. No endoscopic findings suggestive of inflammatory bowel disease, including longitudinal ulcers, cobblestone appearance, skip lesions, or continuous diffuse colitis, were observed. In addition, there was no endoscopic evidence of pseudomembranous colitis, and the patient had no recent history of antibiotic exposure. Although histopathological evaluation of the diverted bowel was not performed, DC was diagnosed based on the characteristic clinical course and endoscopic findings.

**Figure 3:**
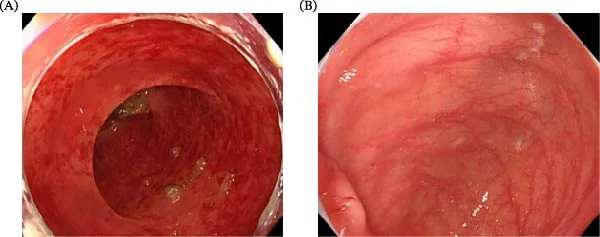
Endoscopic findings before and after treatment. Before treatment (A), marked mucosal atrophy with a high bleeding tendency was observed in a right colon‐dominant excluded colon. After treatment (B), the mucosa appeared almost normal.

On POD48 after ileostomy creation, synbiotic stimulation therapy was initiated using glutamine (14 g/day), Miya‐BM (one tablet containing 20 mg of *C. butyricum*), and Bio‐Three (one tablet containing 2 mg of *Lactomin*, 10 mg of *C. butyricum*, and 10 mg of *Bacillus mesentericus*). Because enema administration might not adequately reach the inflamed right colon, all agents were dissolved together in 100 mL of normal saline and administered anterogradely through the efferent limb of the ileostomy three times per week for 4 weeks. Given the initial narrowing of the intestinal lumen, the solution was administered over ~5 min using a 14‐Fr Nelaton catheter during the first 2 weeks. After sufficient luminal dilatation had been achieved, the catheter was changed to a 21‐Fr Nelaton catheter, and treatment was continued during weeks 3 and 4.

Follow‐up colonoscopy performed on POD77 after ileostomy creation demonstrated near‐complete normalization of the colonic mucosa, including the right colon (Figure 3B). Stoma closure was subsequently performed on POD84 after ileostomy creation. The postoperative course was uneventful, and the patient was discharged on POD 12 after stoma closure.

## 3. Discussion

DC is generally reported to develop within 1 month to 3 years after fecal diversion. Although most patients remain asymptomatic, some patients develop symptoms such as rectal bleeding, abdominal pain, and tenesmus [7]. Alterations in the intestinal microbiota and subsequent depletion of SCFAs—including acetate, propionate, and butyrate—are believed to play major roles in its pathogenesis [2]. SCFAs serve as the main energy source for colonic epithelial cells, and their deficiency leads to disruption of mucosal homeostasis, impaired barrier function, and mucosal injury [2, 3]. Restoration of intestinal continuity by stoma closure is considered the most effective treatment for DC, and symptoms have been reported to improve within ~2 weeks after closure [5]. In contrast, DC generally persists while fecal diversion is maintained, and spontaneous resolution is considered uncommon unless intestinal continuity is restored [1]. In the present case, the stoma had not yet been closed and fecal diversion remained in place; therefore, short‐term improvement of the inflammation through the natural course alone was considered unlikely. Accordingly, given the risk of postoperative complications, we elected to improve the inflammation before stoma closure. Although spontaneous improvement cannot be completely excluded, the marked endoscopic improvement observed 1 month after initiation of treatment is difficult to explain by the natural course alone, suggesting that this treatment may have contributed to the resolution of the inflammation.

The reduction of SCFA‐producing bacteria such as *Bifidobacterium* has been shown to inversely correlate with disease severity [8]. Based on this rationale, with the aim of physiologically improving the intestinal environment and promoting regeneration of the intestinal mucosa, we adopted synbiotic stimulation combining *probiotics* to supplement beneficial bacteria and *prebiotics* to promote their growth and SCFA production. Glutamine was used as a prebiotic, while Miya‐BM and Bio‐Three were used as probiotics. Glutamine, the principal energy source for intestinal epithelial cells, is commonly supplemented at a dose of 10–20 g/day in clinical nutrition to maintain intestinal mucosal integrity [9]. Follow‐up colonoscopy performed 1 month after initiation of therapy demonstrated near‐complete normalization of the mucosal findings. This case suggests that synbiotic stimulation therapy may represent a potential therapeutic option for patients withDC.

Alternative treatments such as 5‐aminosalicylic acid (5‐ASA) or steroid enemas have been reported to be effective, but these require caution due to potential adverse effects and inconsistent efficacy [10, 11].

Thus, synbiotic stimulation may provide a safer and more physiologic treatment option. Furthermore, fecal transplantation has recently been reported as a physiological treatment strategy for DC. Cakir et al. [4] reported that reinfusion of autologous ileal contents through the efferent limb significantly reduced histopathological inflammation, ulceration, goblet cell loss, and crypt abscess formation compared with controls. This approach is thought to contribute to the maintenance of intestinal mucosal homeostasis by restoring exposure of the diverted bowel segment to luminal nutrients, metabolites, and the intestinal microbiota. Similarly, the synbiotic stimulation therapy used in the present case was intended to physiologically improve the intestinal environment. However, unlike fecal transplantation, our approach does not require repeated handling of fecal material and may therefore be a simpler and more feasible treatment option in routine clinical practice. The route of administration may also affect outcomes. While enemas are commonly used, they may fail to deliver agents to the proximal colon. In this case, anterograde administration via the distal stoma limb enabled wider distribution of the agents and was technically simpler. An important remaining question is whether preoperative synbiotic stimulation can reduce postoperative complications after stoma closure in patients with symptomatic DC.

Rodríguez et al. [12, 13] reported the results of the same randomized controlled trial in two separate publications, focusing on the therapeutic effects of probiotic stimulation on DC and POI, respectively. In the analysis using DC as the primary endpoint, probiotic stimulation significantly improved both endoscopic and histological findings [12]. In contrast, the analysis using POI as the primary endpoint demonstrated no significant reduction in the incidence of POI [13]. Notably, patients with anastomotic leakage were excluded from this trial; therefore, the effect of probiotic stimulation on the prevention of anastomotic leakage could not be evaluated.

Lloyd et al. [14] subsequently performed a systematic review and meta‐analysis including three randomized controlled trials and reported that preoperative probiotic stimulation was associated with a reduction in overall postoperative complications, although no significant benefit was observed for POI. Furthermore, when the incidence of anastomotic leakage reported in these randomized trials was reviewed [6, 12, 15], anastomotic leakage occurred in 1 of 117 patients (0.9%) in the intervention group and 5 of 122 patients (4.1%) in the control group. However, because the number of events was small and none of the trials was designed with anastomotic leakage as the primary endpoint, it cannot be concluded that preoperative synbiotic stimulation reduces the incidence of anastomotic leakage. Future prospective studies using anastomotic leakage as the primary endpoint are warranted to further investigate this issue.

Diversion colitis is generally considered to be effectively treated by restoration of intestinal continuity through stoma closure. In the present case, marked improvement in both endoscopic and clinical findings was observed after synbiotic stimulation administered through the efferent limb of the ileostomy, followed by successful stoma closure. Although the efficacy of this treatment cannot be established based on a single case, synbiotic stimulation via the efferent limb may represent a therapeutic approach that warrants further investigation for DC.

## Funding

No funding was received for this manuscript.

## Consent

Written informed consent was obtained from the patient for publication of this case report and the accompanying images.

## Conflicts of Interest

The authors declare no conflicts of interest.

## Data Availability

The data that support the findings of this study are available upon request from the corresponding author. The data are not publicly available due to privacy or ethical restrictions.
